# A Novel Fire Risk Assessment Approach for Large-Scale Commercial and High-Rise Buildings Based on Fuzzy Analytic Hierarchy Process (FAHP) and Coupling Revision

**DOI:** 10.3390/ijerph18137187

**Published:** 2021-07-05

**Authors:** Yijie Wang, Linzao Hou, Mian Li, Ruixiang Zheng

**Affiliations:** 1 University of Michigan-Shanghai Jiao Tong University Joint Institute, Shanghai Jiao Tong University, Shanghai 200240, China; yijiewang@sjtu.edu.cn (Y.W.); houlinzao@sjtu.edu.cn (L.H.); mianli@sjtu.edu.cn (M.L.); 2 Department of Automation, Shanghai Jiao Tong University, Shanghai 200240, China

**Keywords:** fire risk assessment, large-scale commercial and high-rise buildings, FAHP, coupling revision, public safety

## Abstract

In recent years, much more emphasis than before has been placed on fire safety regulations by the local and central authorities of China, which makes fire risk assessments more important. In this paper we propose a new fire risk assessment approach for large-scale commercial and high-rise buildings that aims to evaluate the performances of their fire safety systems; this should improve the fire risk management and public safety in those buildings. According to the features of large-scale commercial and high-rise buildings, a fire-risk indexing system was built, and based on it we established a scientific fire risk evaluation system. To this end, the fuzzy analytic hierarchy process (FAHP) was used to assign a reasonable weight to each fire risk factor in the evaluation system. In addition, we revised the original scores by analyzing the coupling relationships among the fire risk factors. To validate our system, we selected 11 buildings in Shandong province and collected their fire safety data. Then, we calculated the final scores for the fire safety management of those buildings, and the results show that: (1) our fire risk evaluation system can assign reasonable weights; (2) the proposed evaluation system is comprehensive and has strong interpretability, since it exploits the coupling relationships among the risk factors. The novelty of the proposed approach lies in that it integrates opinions from multiple experts and utilizes coupling relationships among the factors. Further, the feedback from the approach can find not only the weaknesses in fire risk management, but also the potential causes of fires. As a result, the feedback from our assessment can assist the safety chiefs and inspectors with improving fire risk management.

## 1. Introduction

Fire is a common risk in our daily lives. In recent years, the annual average number of fires reported in China has exceeded 100,000, causing more than 2000 deaths and over one billion US dollars in losses each year [1,2]. Therefore, fire risk management is very essential for ensuring the safety of people’s lives and property. Among all kinds of buildings, large-scale commercial and high-rise buildings are the most representative ones located in cities with high population densities and are tied closely to economic development and people’s lives. Therefore, plenty of laws, regulations and relevant provisions about those two kinds of buildings have been published by the government to regulate the fire safety management of those buildings [3,4]. In order to enhance the fire safety management of those buildings, a well-designed assessment that fully considers the features of those two types of buildings is demanded. A scientific assessment can motivate the safety chiefs of the buildings (the safety chief is the person in charge of fire safety in a building) to improve their fire security systems so as to ensure fast responses to conflagration and to improve self-help ability against the disaster, which should protect the lives and property of the residents.

The current assessment of fire safety management relies mostly on professional fire maintenance staff and manually-conducted statistics, analysis and inspection [5]. In such a manner, the data utilization is ineffective because: Firstly, the manual data collection and analyses based on maintenance reports and annual assessment reports of fire management are very time consuming and sometimes inaccurate. Secondly, due to the lack of systematic approaches to evaluating the fire safety management of buildings, the current data collection procedure is usually unscientific and inconsistent. Last but not the least, currently many data are collected but not used for any assessments. Hence, the potential of data mining and analysis is huge, and once performed, the development of the fire prevention and control will be furthered.

To tackle the problems mentioned above, we propose a novel fire risk assessment approach based on the policy documents published by the Chinese Ministry of Emergency Management. The major goal of this work was to design a comprehensive, reliable and expert-knowledge-based fire risk evaluation system for large-scale commercial and high-rise buildings. To that end, this paper first investigated the national standards of fire safety management, the monthly maintenance reports and the annual fire assessment reports. Based on those documents, the assessment considered multiple perspectives, such as the basic features of those two types of buildings, fire emergency rescue capabilities and the maintenance of firefighting facilities. We present our fire risk evaluation system based on the proposed approach, and there are two highlights of this evaluation system. First, among many multiple-criteria decision analysis (MCDA) methods which can be applied to assign weights in risk assessment, such as the analytic hierarchy process (AHP) [6], the technique for order preference by similarity (TOPSIS) [7,8] and VlseKriterijumska Optimizacija I Kompromisno Resenje (VIKOR) [9,10], we chose the fuzzy analytic hierarchy process (FAHP) method for our approach, which is the fuzzy extension of the AHP method and integrates the advantages of the AHP method. The AHP is commonly used for assessment purposes [11]. The AHP method can assign weights by solving eigenvalue problems [12] while keeping consistency of the relative importance between each pair of factors [6]. Moreover, in order to synthesize the knowledge from different experts in the field of fire safety management, the FAHP was finally chosen to assign weights to the categories and fire risk factors in the proposed fire risk evaluation system [13,14]. Second, we analyzed the coupling relationships among the fire risk factors based on the national standards. Then, we utilized those coupling relationships to revise the original scores (provided by third parties) of the fire risk factors to make the assessment comprehensive and interpretable [15,16]. We used the proposed fire risk evaluation system to assess 11 buildings in Fangzi (located in Weifang, Shandong province of China), and the results show that our approach is effective. The proposed fire risk evaluation system is valuable to society, since it can be useful for both the supervision department of the government and the safety chiefs of the buildings. To be specific, the supervision department can use the system to assess the capabilities of fire safety management of buildings, and then conduct targeted inspection actions. Meanwhile, the safety chief of a building can use the system to find out the defects of its fire security system and then carry out self-rectification.

The main contribution of this work is to propose a novel fire risk assessment approach for large-scale commercial and high-rise buildings. The proposed approach uses the FAHP to assign the weights for categories and factors during the fire risk assessment by integrating knowledge of multiple experts. Therefore, the FAHP makes the weights more scientific and reliable. Moreover, the proposed approach revises the scores of factors based on the coupling relationships between the factors. By considering not only the factors with low scores but also those coupled factors with awful performance, the assessment result can help the safety chiefs and inspectors efficiently identify the most urgent risk factors to improve the fire safety management. One innovation of this work is to provide a way to make the fire risk assessment more objective by combining the knowledge and experience of multiple experts. The other innovation is to consider the cause and effect coupling relationship between each pair of factors in a fire-risk indexing system such that both the safety chiefs and the inspectors can efficiently improve the weaknesses in the fire safety management of a building. For the follow-up development of a fire risk assessment, an important topic in the public health discipline, our work provides assistance. To be specific, the proposed work offers convincing weights assigned by multiple experts, and the coupling relationships find out all the weaknesses in fire safety management to reduce the potential financial losses and deaths resulting from fires. Regarding the risk assessment, this approach provides a meaningful way to assign weights based on a risk indexing system. This work also points out that people should consider the cause and effect relationships in the risk indexing system. Such relationships usually exist in the risk assessment and the real world. However, the relationships are usually ignored, and some factors leading to potential problems are not rectified after the risk assessment. After some time, those factors will cause other factors to become significant problems as well. Therefore, the coupling relationship should be considered in risk assessment to solve the above problem. The novelty of our paper is that to the best of the authors’ knowledge, the proposed approach is the first to originally utilize both the FAHP method and the coupling relationships in the fire-risk indexing system. Exploiting the coupling relationships in the fire-risk indexing system is our original scientific concept. Based on this original concept, the coupling revision method is proposed to revise the scores to make fire risk assessments much more scientific and reasonable. Moreover, the feedback of this approach can find not only the weaknesses in fire risk management, but also the potential causes of fires. Thus, it can help the safety chiefs and inspectors to improve the fire risk management of buildings.

The rest of the paper is organized as follows. We introduce the related works in Section 2. The details of our methodology are presented in Section 3, including the fire-risk indexing system, the FAHP and the revised scores based on coupling relationship analysis. In the same section, the pseudo-code is provided to illustrate the approach. In Section 4, 11 large-scale commercial and high-rise buildings have their fire safety management assessed using the proposed fire risk evaluation system. In Section 5, first, we verify the advantages of the FAHP and coupling revision by comparing our approach with previous ones. Then, we present interviews of experts whom we asked about those 11 buildings’ fire risk assessment results to verify the assessment quality. We use an example to illustrate how the assessment scores reflect the reality and lead the safety chiefs and the inspectors to improve the fire risk management of each building. Moreover, we discuss the possibility of generalizing the proposed approach for other kinds of buildings and the future work at the end of that section. The conclusion is given in Section 6.

## 2. Related Works

Fire risk assessments of buildings are essential for fire prevention and control, and scholars have made significant efforts in this direction. In the literature, one of the most fundamental and commonly used approaches is fire risk indexing (FRI) [17,18]. This approach first designs a fire-risk indexing system containing all possible attributes that may cause fires or increase the risk, together with reasonable weights that are assigned to those factors. Then by assigning a score to each factor, a weighted final score (or the fire-risk indexing) can be calculated to quantitatively assess the fire risk of the building. This method is flexible and adaptive to different types of buildings, such as historic and old buildings [19], high-rise buildings [20] and factories [21]. By adjusting the factors in the fire-risk indexing system according to specific type of building, the method can be applied to the assessment of any building [22]. Generally speaking, the fire-risk indexing system, weights and scores are the three major components of the FRI method. The work in [19] provides a detailed fire-risk indexing system designed for old buildings, and the fire-risk indices of all buildings are visualized on a map of the corresponding region. The indexing system and weights are defined subjectively in that paper, which is flexible but undermines the validity of FRI. In [22], the authors focused on FRI and compared several fire-risk indexing systems. They restricted the sources of fire risk factors to national authoritative documents or building codes. They also analyzed the influences of all factors on the fire risk and selected important ones to form a more scientific indexing system.

Besides the analysis of indexing systems, other studies considering weight assignment and score determination in FRI are also essential. In order to assign weights in the FRI system, numerous MCDA methods can be applied. For example, AHP [6] is a kind of MCDA method that uses pairwise comparisons of criteria in the comparison matrix to determine which one is more important [11] in a hierarchical system [23]; then the weights are obtained by solving the eigenvalue and eigenvector problem for the comparison matrix. TOPSIS [7,8] is a MCDA method that compares the geometric distance (e.g., Euclidean distance) among each alternative and the ideal alternatives; the chosen alternative should have the minimal geometric distance from the positive ideal solution and the maximal geometric distance from the negative ideal one. VIKOR [9,10] is similar to TOPSIS such that the alternative is selected by the closeness to the ideal solutions, and the closeness is measured in different ways. The Complex Proportional Assessment (COPRAS) was proposed in [24,25] to evaluate the superiority of any one alternative over another, and made it possible to compare alternatives [26]. The alternatives are ranked and evaluated by their importance and degrees of utility [27]. The Preference Ranking Organization METHod for Enrichment Evaluation (PROMETHEE) II is based on the pair-wise comparisons of alternatives and each selected criterion; this method requires two additional inputs—one is the weights of the criteria and the other is a decision-maker’s preference function, which is used to compare the alternatives [28,29]. For TOPSIS, VIKOR and COPRAS, the weights can be obtained by several methods, such as the entropy method (EM) [30] and the mean weight (MW) [31,32]. For PROMETHEE II, the weights are determined by using the Delphi method [33]. In order to choose the proper method, reference [34] performed a comparative study for the above MCDA methods to indicate that not only the method itself but also the method of normalization and other parameters should be carefully selected.

In our application, we chose to use the FAHP method, which is the fuzzy extension of the AHP method and integrates the following advantages of the AHP: (1) the AHP is a widely used MCDA method in many applications and problems according to the survey results in [11]; (2) psychologists have discovered that it is easier to make judgments on a pair of alternatives at a time than simultaneously on all the alternatives [12]; (3) the weights can be obtained by solving the eigenvalue problem for the comparison matrix while maintaining the relative importance for each pair of factors. In [35], the risk evaluation part introduced the AHP for determining the weights of risk factors. It generated more valid weights according to the relative importance of the factors. However, the AHP is a subjective method and the importance comparison is based on the opinion of only one expert. To overcome this problem, we designed a new questionnaire to consult multiple experts for weight determinations and used the FAHP method to summarize the opinions from those experts. The FAHP is the fuzzy extension of the AHP method, and FAHP methods with triangle and trapezoidal fuzzy numbers were the first attempts at fuzzy extensions. For example, [36] presented the idea of using weights given by multiple experts to generate fuzzy values. The usage of trapezoidal fuzzy numbers to determine reasonable weights in many other applications is found in [37]. In [38], the intuitionistic fuzzy analytic hierarchy process (IFAHP) was proposed based on intuitionistic fuzzy values. This method finds a new way to check the consistency of preferences and introduces an automatic procedure to repair the inconsistent cases. In [39], the authors proposed a consensus-based fuzzy extension of the AHP method for selecting and evaluating suppliers in an incomplete fuzzy preference relations (IFPRs) environment utilizing $T_{L}$-transitivity. In our application, we chose to use the FAHP with triangle fuzzy numbers (TFNs) to combine the opinions of multiple experts and assign weights. For a TFN, its medium value represents the most possible value, and the two other values represent the smallest possible and the largest possible values of the relative importance in a pair-wise comparison, respectively. A TFN makes building managers and engineers feel comfortable with providing estimates in terms of most likely values and ranges of possibility. Therefore, the FAHP with TFN is suitable for assessing building risks, including the fire risks in buildings [36,40].

After assigning the weights, the computation of scores in the assessment with FRI should be studied. Reference [41] introduced a new perspective of dynamic correlations among factors, which were defined as coupling relationships. In the fire-risk indexing system, those coupling relationships indicate how the factors are affected by each other, so we can obtain both the current performance of a factor and its potential trends. All those studies improved the basic FRI in terms of the indexing system, weights and scores, making the method more complete and useful.

Apart from FRI, there are also attempts at risk assessments using other methods. For example, [42] applied logistic regression and a deep neural network to evaluating fire risk based on historical fire occurrence data. Although the risk prediction could be reasonable, those models cannot provide useful feedbacks to help with practical improvements. The authors of [43] constructed scenario clusters related to fire behaviors such as ignition, growth and firefighting to evaluate the fire risk. However, the scenarios may vary with different buildings, which makes it difficult to generalize this method. The work in [44] derived a flow model of passengers inside a metro based on the multi-velocity floor field cellular automaton (FFCA) model to create a dynamic risk assessment, which focused more on human behaviors in the building.

Considering all the methods discussed above, FRI is still the most important and intuitive one for the fire risk assessment. It can not only predict the fire risk but also provide practical instructions for risk reduction, since the method is derived from practical fire risk factors. Therefore, our approach concentrates on improving the weight assignments and exploiting coupling relationships among the factors in the FRI method, thereby bettering the assessment scheme.

## 3. Methodology

**Figure 1:**
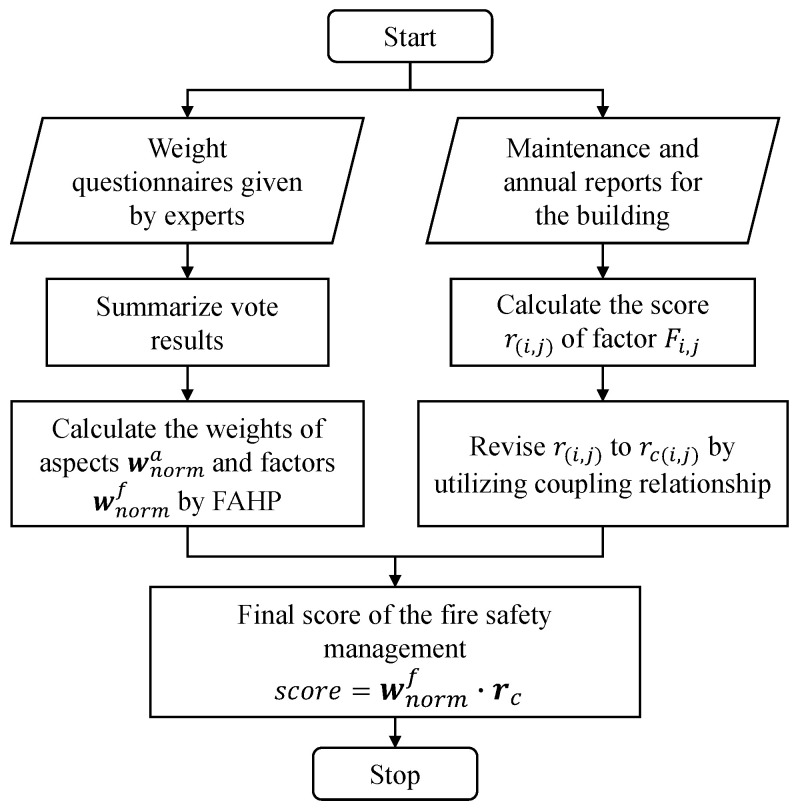
The overall framework for our fire risk assessment approach.

In this section, we introduce our methodology in the following steps: (1) we first establish a fire-risk indexing system, based on which we can assess the management performance; (2) with the fire-risk indexing system designed, we provide a weight for each factor using the FAHP method; (3) according to the coupling relationships among the fire risk factors, we integrate those coupling relationships into the fire-risk indexing system and revise the score of each factor by utilizing the quantified coupling extent. The second and third steps are the major contents of the proposed fire risk assessment approach. The overall framework of our methodology for fire risk assessments is shown in Figure 1.

### 3.1. The Fire-Risk Indexing System

To conduct a fire risk assessment, we must have a fair and comprehensive indexing system. In this system, all factors are fully relative to the features of large-scale commercial and high-rise buildings based on the national standards on fire safety management, the maintenance reports from third parties and the annual fire management assessment reports. After investigating the above contents, we created a fire-risk indexing system with a tree structure, as shown in Table 1. As shown in the tree structure, there are 4 categories and 18 factors. Those factors were chosen based on the national standards for fire safety management, the maintenance reports from third parties, the annual fire management reports and the consultations with multiple experts in the firefighting field. Combining the above information, the factors were finally determined by considering the modern requirements of fire control. Within the 18 factors there are 89 items, and those items involve multiple considerations for a fire risk assessment. The explanations of the 18 factors are as follows:

- Building legitimacy: The building should have legitimate records in the corresponding administrative office.
- Fire safety regulation: The safety chief of the building should construct complete regulations for fire safety management.
- Fire safety operation standard: The implementation and operation of all the regulations and facilities should be standardized.
- Fire prevention patrol: The property management office should arrange a team to exercise a daily fire prevention patrol and set up a patrol record.
- Publicity and education: There should be posters in conspicuous places emphasizing basic fire safety knowledge.
- Power supply and distribution: The power supply and distribution should work normally during emergencies.
- Automatic fire alarm system: The building should be equipped with automatic smoke detectors and fire alarm systems.
- Automatic water spraying system: The building should be equipped with automatic water spraying fire extinguishing systems.
- Smoke exhaust system: The building should have a suitable smoke exhaust design and facilities.
- Fire separation facility: The building should have fire separation facilities including fireproof rolling shutter doors and fire compartmentation areas.
- Fire elevator: The building should be equipped with enough fire elevators.
- Emergency lighting: Emergency fire lights should keep working during power failures.
- Emergency broadcast: The building should be equipped with emergency broadcast facilities.
- Water supply facility: Water supply is essential for fire extinguishing and should be checked regularly.
- Self-rectification: All the defects found during inspections should be rectified within a reasonable timeframe.
- Firefighting ability: Firefighting ability contains the following aspects: the building should have adequate fire distinguishing facilities; there should be several teams that know basic firefighting operations; and the evacuation drills should be organized regularly.
- Electricity and gas safety: The use of electricity and gas should be checked regularly to ensure safety.
- Evacuation facilities: The evacuation facilities should not be blocked.

Those 18 factors can be summarized into 4 categories: fire control management, facility maintenance, firefighting and rescue, and potential risk inspection. The fire-risk indexing system in our method is listed in Table 1.

### 3.2. Weight Distribution with the FAHP

In this subsection, we introduce a proper method of weight distribution among the categories and factors via the FAHP. To that end, we first designed a questionnaire for the categories and factors in our fire-risk indexing system in order to collect experts’ knowledge on the importance of each category and factor. Then, based on the questionnaire results from multiple experts, we generated a tuple for each category/factor to represent the overall opinion of all the experts and obtain the fuzzy matrix. Finally, based on the fuzzy matrix, we created the weight distribution for our fire-risk indexing system. The corresponding details will be introduced as follows.

#### 3.2.1. Design Questionnaire

In this part, according to [36], we show our questionnaire used to collect the experts’ opinions efficiently and prepare for further analysis.

Table 2 consists of two parts, the upper part evaluates the influences of *M* categories (M=4 in our scenario); the lower part is for the factors belonging to the same categories. The value of importance for each factor or category is an integer between 1 and 9: 1 means that the corresponding factor has the least importance in the same category or the category has the least importance in the fire-risk indexing system; on the contrary, 9 means that the factor/category has the most importance. By collecting the values of importance for each category/factor using Table 2, the opinions of all experts’ were compiled. The procedures for the synthesis are introduced in the following subsection.

#### 3.2.2. Preprocessing

The preprocessing procedure was used to generate the fuzzy matrix from the raw data, which were collected from the questionnaire shown in Table 2. The preprocessing procedure for categories and factors had the same mechanism. In the following discussion, we show the details for categories. Factors were treated similarly.

During the preprocessing, if we invite $n_{e}$ experts for the fire risk assessment and there are *n* categories in the proposed fire-risk indexing system, the overall questionnaire results can be denoted as a vector $T=\left(T_{1},T_{2},\cdots ,T_{k},\cdots ,T_{n_{e}}\right),T_{k}\in {\left[t_{k,q}^{\left(i\right)}\right]}_{n\times 9},t_{k,q}^{\left(i\right)}\in \left\{0,1\right\},i\in \left\{1,2,\cdots ,n\right\},q\in \left\{1,2,\cdots ,9\right\}$, where the matrix $T_{k}$ represents the *k*th expert’s questionnaire results in Table 2 and $t_{k,q}^{\left(i\right)}=1$ represents that the *k*th expert assigned *q* to the importance of the *i*th category.

The matrix $S\in {\left[s_{iq}\right]}_{n\times 9},i\in \left\{1,2,\cdots ,n\right\},q\in \left\{1,2,\cdots ,9\right\}$ is the overall results from all experts and can be obtained by:

(1) $$S=\sum_{k=1}^{n_{e}}T_{k},$$

where $s_{iq}$ is the number of experts who assigned *q* to the importance of the *i*th category.

For the *i*th row of *S*, according to the value of the element $s_{iq}$, we can use the tuple $\left(l^{\left(i\right)},m^{\left(i\right)},u^{\left(i\right)}\right)$ to summarize the opinions of all the experts, where the smallest index of the nonzero element is denoted as $l^{\left(i\right)}$, the index of the largest element is denoted as $m^{\left(i\right)}$ and the largest index of the nonzero element is denoted as $u^{\left(i\right)}$. In order to compare the importance for each pair of factors, the fuzzy matrix $F={\left[f_{ab}\right]}_{n\times n}$ is used. The procedure to generate fuzzy matrix *F* can be summarized with the following steps:

1. $f_{aa}=\left(1,1,1\right),a=1,2,\cdots ,n$;
2. for a,b=1,2,⋯,n,a≠b:
  - if $u^{\left(a\right)}=l^{\left(b\right)}$ and $l^{\left(a\right)}=u^{\left(b\right)}$, then $f_{ab}=\left(1,1,1\right)$;
  - if $m^{\left(a\right)}>m^{\left(b\right)}$, then
    $$f_{ab}=\left(\max\left(\mathrm{round}\left(\frac{m^{\left(a\right)}}{m^{\left(b\right)}}\right)-2,1\right),\max\left(\mathrm{round}\left(\frac{m^{\left(a\right)}}{m^{\left(b\right)}}\right),1\right),\min\left(\mathrm{round}\left(\frac{m^{\left(a\right)}}{m^{\left(b\right)}}\right)\;+\;2,\;9\right)\right);$$
  - else $f_{ab}=\left(\frac{1}{\min(\mathrm{round}\left(\frac{m^{\left(b\right)}}{m^{\left(a\right)}}\right)\;+\;2,\;9)},\frac{1}{\max(\mathrm{round}\left(\frac{m^{\left(b\right)}}{m^{\left(a\right)}}\right),\;1)},\frac{1}{\max(\mathrm{round}\left(\frac{m^{\left(b\right)}}{m^{\left(a\right)}}\right)\;-\;2,\;1)}\right)$.

Here, the function max(·,·) is used to obtain the maximum of arbitrary two real numbers; round(·) is used to obtain the nearest integer of an arbitrary real number. After the above procedure, the element $f_{ab}$ of the fuzzy matrix *F* is called the triangular fuzzy number (TFN) and can be denoted as $f_{ab}=\left(l_{ab},m_{ab},u_{ab}\right)$. The possible values for all TFN are collected in Table 3 [40].

In fuzzy set theory [45], such TFN tuples in Table 3—e.g., (l,m,u)—are used to construct the fuzzy set *M* by the membership function $\mu_{M}:X\to \left[0,1\right]$ measuring the degree of membership of an arbitrary number *x* in *X* that belongs to the fuzzy set *M*. The membership function $\mu_{M}$ can be shown as follows [36,40,46].

(2) $$\mu_{M}\left(x\right)=\left\{\begin{array}{cc} \frac{1}{m\;-\;l}x-\frac{l}{m\;-\;l}, & x\in [l,m]\\ \frac{1}{m\;-\;u}x-\frac{u}{m\;-\;u}, & x\in [m,u]\\ 0, & \mathrm{otherwise} \end{array}\right.$$

**Figure 2:**
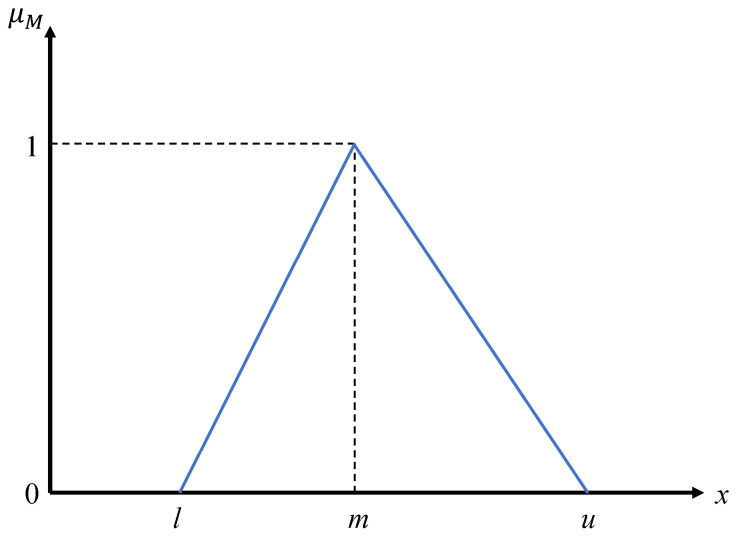
The membership function of triangular fuzzy number (TFN).

The following Figure 2 visualizes the membership function.

After obtaining the fuzzy matrix *F*, we have finished the preprocessing procedure; the related pseudo-code is shown as Algorithm 1.
**Algorithm 1:** Generating the fuzzy matrix.
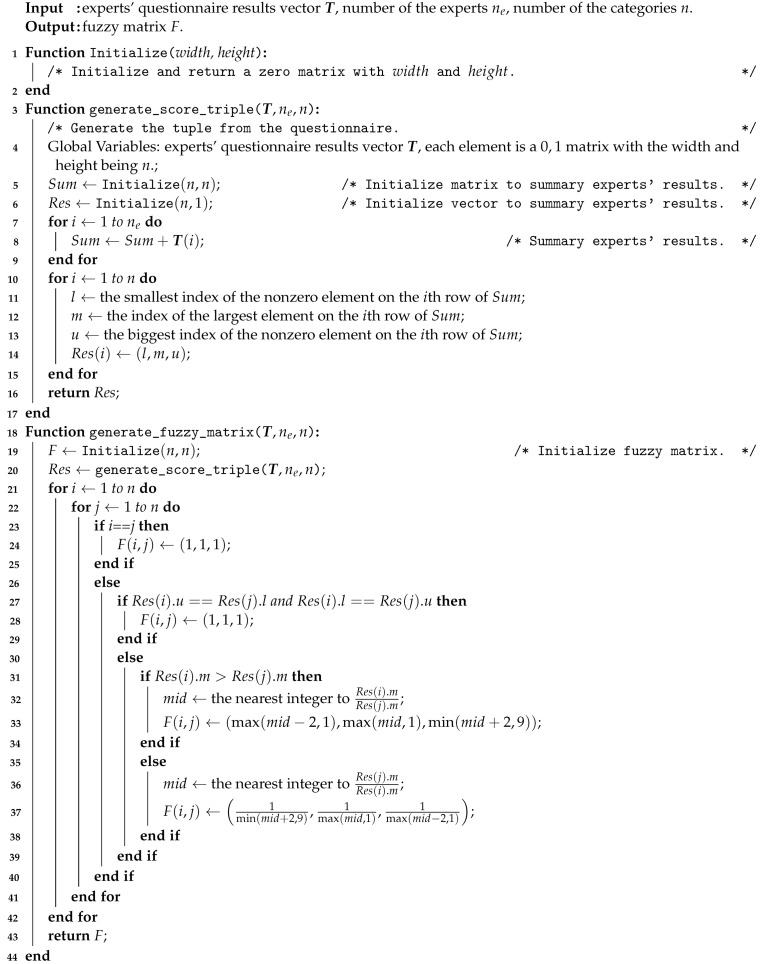


#### 3.2.3. Weight Distribution with the Fuzzy Analytic Hierarchy Process (FAHP)

**Figure 3:**
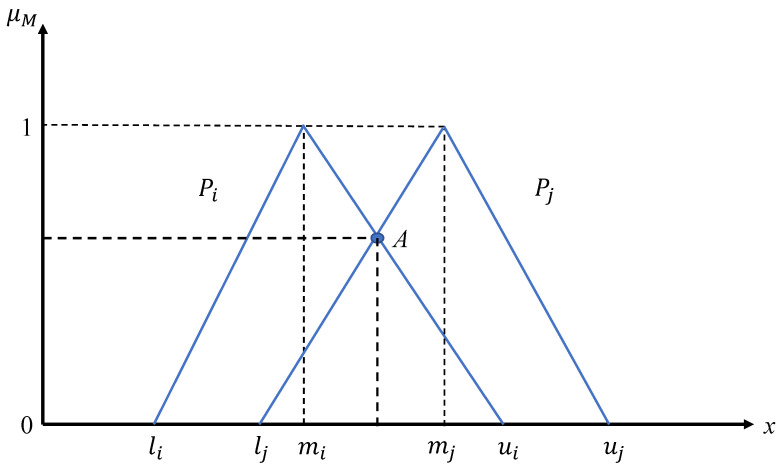
The comparison between two triangular fuzzy numbers.

The FAHP distributes weights among all categories and all factors belonging to the same category. In the following we show details of distributing weights among categories; once understood, weights among factors can be distributed similarly. The steps are summarized as follows [36,40,46]:

1. After obtaining the fuzzy matrix $F={\left[f_{ab}\right]}_{n\times n}$, the value of the fuzzy synthetic extent of the *i*th category should be
  (3) $$\begin{array}{cc} P_{i} & =\sum_{b=1}^{n}f_{ib}⊗{\left(\sum_{a=1}^{n}\sum_{b=1}^{n}f_{ab}\right)}^{-1}\\ \; & =\left(\sum_{b=1}^{n}l_{ib},\sum_{b=1}^{n}m_{ib},\sum_{b=1}^{n}u_{ib}\right)⊗{\left(\sum_{a=1}^{n}\sum_{b=1}^{n}l_{ab},\sum_{a=1}^{n}\sum_{b=1}^{n}m_{ab},\sum_{a=1}^{n}\sum_{b=1}^{n}u_{ab}\right)}^{-1}\\ \; & =\left(\sum_{b=1}^{n}l_{ib},\sum_{b=1}^{n}m_{ib},\sum_{b=1}^{n}u_{ib}\right)⊗\left(\frac{1}{\sum_{a=1}^{n}\sum_{b=1}^{n}u_{ab}},\frac{1}{\sum_{a=1}^{n}\sum_{b=1}^{n}m_{ab}},\frac{1}{\sum_{a=1}^{n}\sum_{b=1}^{n}l_{ab}}\right)\\ \; & =\left(\frac{\sum_{b=1}^{n}l_{ib}}{\sum_{a=1}^{n}\sum_{b=1}^{n}u_{ab}},\frac{\sum_{b=1}^{n}m_{ib}}{\sum_{a=1}^{n}\sum_{b=1}^{n}m_{ab}},\frac{\sum_{b=1}^{n}u_{ib}}{\sum_{a=1}^{n}\sum_{b=1}^{n}l_{ab}}\right), \end{array}$$
  where the operator ⊗ means the element-wise multiplication. We denote the elements of $P_{i}$ as $\left(l_{i},m_{i},u_{i}\right)$.
2. Construct the comparison matrix $U={\left[\mu_{ab}\right]}_{n\times n}$ according to the fuzzy synthetic extents, and each element $\mu_{ab}$ is obtained by the following steps:
  - If a=b, then $\mu_{ab}=\infty$;
  - If a≠b, then $\mu_{ab}$ is determined by the values of $P_{a}=\left(l_{a},m_{a},u_{a}\right)$ and $P_{b}=\left(l_{b},m_{b},u_{b}\right)$ with the following equation:
    (4) $$\mu_{ab}=\left\{\begin{array}{cc} 1, & m_{b}\leq m_{a}\\ 0, & u_{a}\leq l_{b}\\ \frac{l_{b}\;-\;u_{a}}{(m_{a}\;-\;u_{a})\;-\;\left(m_{b}\;-\;l_{b}\right)}, & \mathrm{otherwise} \end{array}\right.$$
  Here, $\mu_{ab}$ represents the membership degree where the tuple $\left(P_{a},P_{b}\right)$ belongs to the fuzzy set
  M:={categorybismoreimportantthancategorya}.
  For the third condition in the Equation (4), it reflects the value of the intersection point, which is shown as point *A* in the Figure 3.
3. Then every weight vector w for every category is obtained as:
  (5) $$\begin{array}{cc} w & =[w_{1},w_{2},\cdots ,w_{n}]\\ \; & =\left[\min_{b=1,2,\cdots ,n}\mu_{1b},\min_{b=1,2,\cdots ,n}\mu_{2b},\cdots ,\min_{b=1,2,\cdots ,n}\mu_{nb}\right]. \end{array}$$
4. The final weight factor should be normalized. The normalization is done differently for categories and factors, which is summarized as follows:
  - For categories, perform:
    (6) $$w_{norm}^{a}=\left[\frac{w_{1}}{\sum_{i=1}^{n}w_{i}},\frac{w_{2}}{\sum_{i=1}^{n}w_{i}},\cdots ,\frac{w_{n}}{\sum_{i=1}^{n}w_{i}}\right].$$
  - For factors, assume the *i*th category has normalized weight $w_{norm}^{a,i}$; then for the $N_{i}$ factors in this category, perform:
    (7) $$w_{norm}^{f,i}=\left[\frac{w_{1}}{\sum_{i=1}^{N_{i}}w_{i}},\frac{w_{2}}{\sum_{i=1}^{N_{i}}w_{i}},\cdots ,\frac{w_{N_{i}}}{\sum_{i=1}^{N_{i}}w_{i}}\right];$$
    for the weights of all the factors, perform:
    (8) $$w_{norm}^{f}={\left[w_{norm}^{f,i}\cdot w_{norm}^{a,i}\right]}_{i=1}^{n},$$
    where the dimension of the vector $w_{norm}^{f}$ is $\sum_{i=1}^{n}N_{i}$.

**Figure 4:**
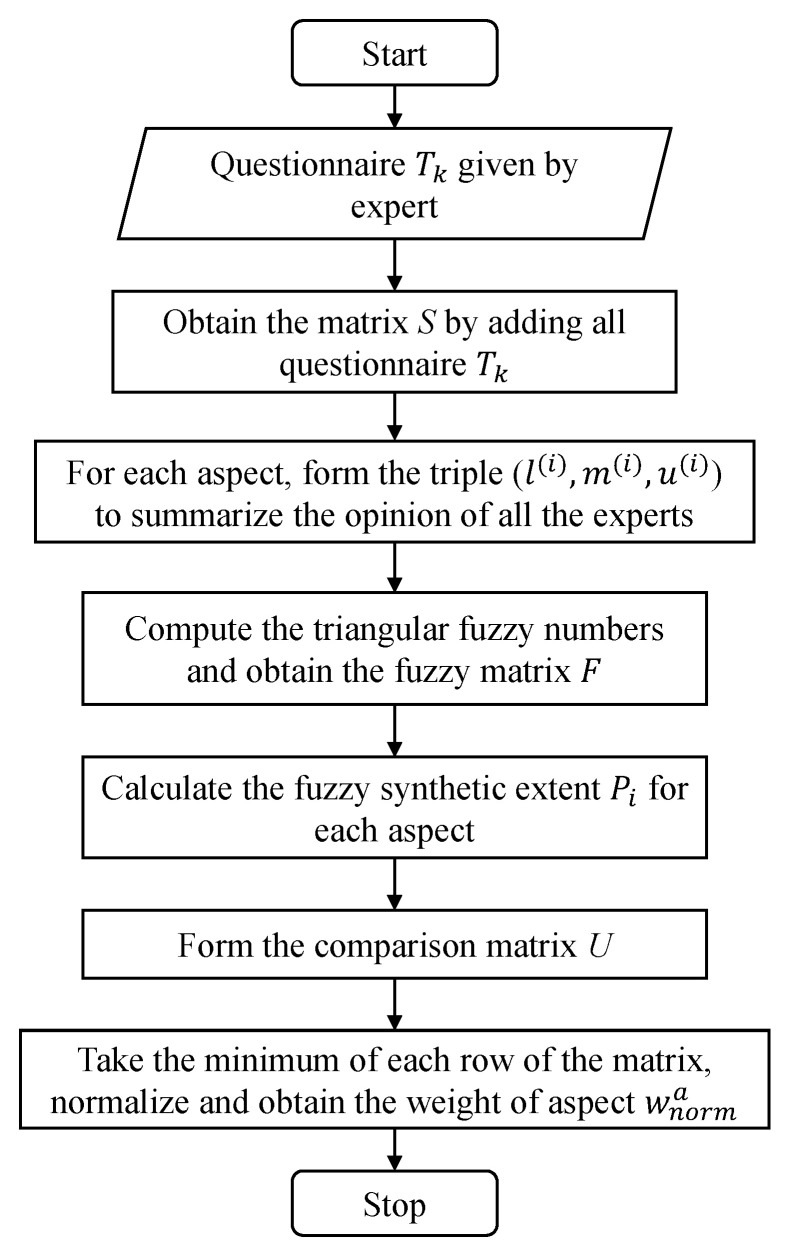
The flow chart of the FAHP algorithm.

The overall steps are summarized in Figure 4; the corresponding pseudo-code is shown as Algorithm  2.
**Algorithm 2:** The FAHP.
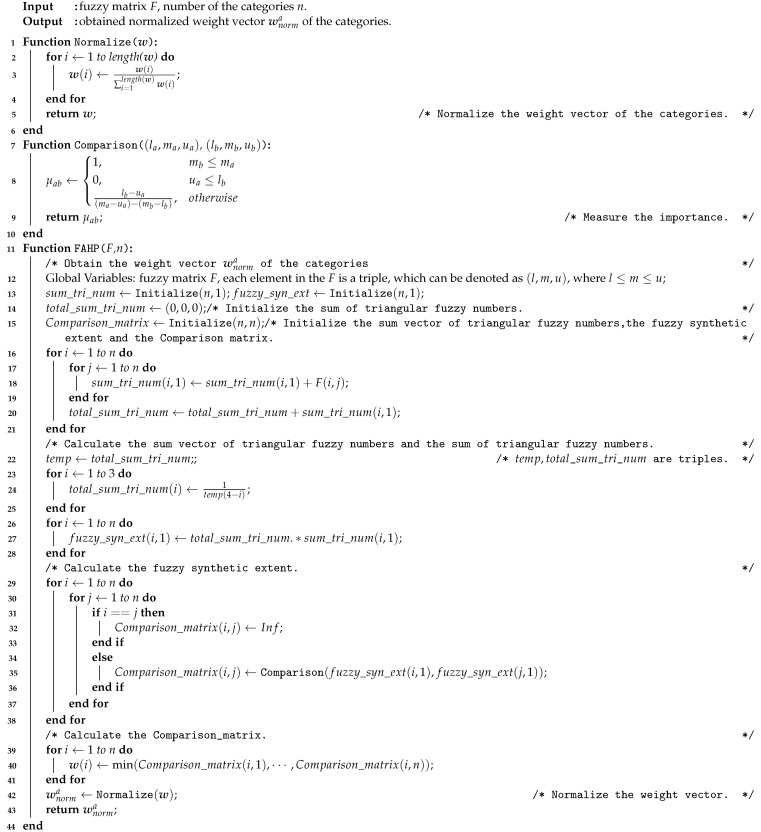


### 3.3. Score Revision Based on Coupling Relationship Analysis

At this point, we have shown how we designed the fire-risk indexing system and assigned the weights to the categories and factors. The next step was to score each factor according to the actual situation of the building, and then the final weighted fire risk score could be obtained. Usually, the scoring process is rather independent, since the inspectors only consider fixed items for each factor. For example, while scoring the factor “firefighting ability”, the inspector usually considers the allocation of firefighting facilities and evacuation exercises. While scoring the factor “publicity and education”, the general knowledge of employees and residents about fire safety is evaluated. However, in reality, those risk factors are not totally independent. Usually the lack of education of employees or residents about firefighting will diminish the ability to firefight. Such a cause and effect relationship is not fully considered when the inspector is scoring each factor. Therefore, we introduce the concept of coupling in this work. The coupling concept is defined as: if a certain category/factor has bad performance and it has side-effects on the performance of another category/factor, then these two categories/factors are regarded as “coupled”. Such a coupling relationship is directional, and can be one-to-one, one-to-many or many-to-one. In our coupling relationship analysis, the cause and effect relationship was considered by introducing a coupling coefficient for each cause and effect pair to describe the coupling between two factors. Then, the score given by the inspector of the affected factor was revised.

**Figure 5:**
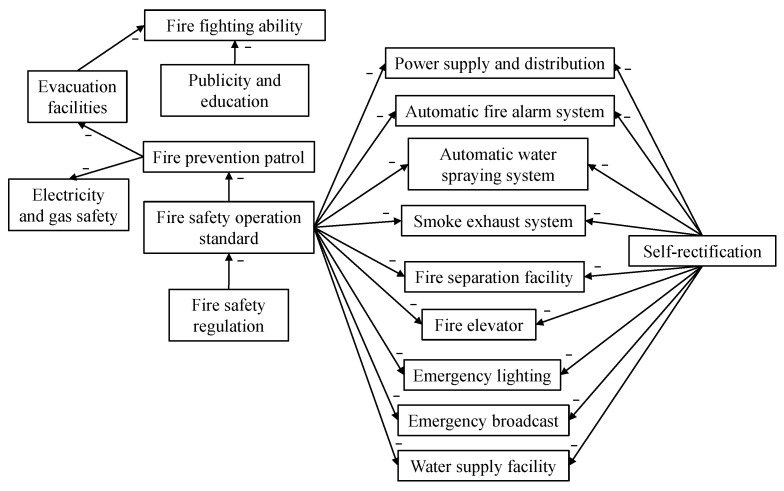
The coupling relationships among factors.

The coupling revision process contained the following procedures. First of all, we analyzed all the factors and figured out all the cause and effect pairs. In the proposed fire-risk indexing system, there are 24 such pairs, and we summarize them in Figure 5.

In Figure 5, each arrow represents a cause and effect pair. The direction of the arrow points from the causal factor to the affected factor, and a minus sign above an arrow means that the cause will lower the performance of the affected factor, which is explained later. To better understand those relations, we use the following example. Consider all the pairs between “self-rectification” and “fire safety facilities”. According to the regulations of the government, the property management department of each building should ask professional maintenance companies to check all fire safety facilities regularly, and take necessary self-rectification measures. Therefore, the factor “self-rectification” is directly related to the performances of all those facilities, and unqualified self-rectification will lead to bad performances by facilities. This is how cause and effect coupling relationship works. All other pairs in Figure 5 can be explained in similar ways.

After determining all the coupling relationship, we can calculate the coupling coefficient for each pair to quantify the extent of the influence in this relation. We referred to the work in [41] and found a suitable formula for a coupling coefficient as follows:

(9) $$c_{y\;-\;x}={\left\{\frac{r_{y}r_{x}}{{\left(\frac{r_{y}\;+\;r_{x}}{2}\right)}^{2}}\right\}}^{5}$$

where $r_{x}$ and $r_{y}$ are scores given by the inspector to the two factors *x* and *y*, respectively, involved in this coupling relationship, where the factor *x* is the cause and the factor *y* is the effect.

Next, after obtaining the coupling coefficients, we should consider how they could be used to revise the original scores. We only consider the situation that a bad cause will undermine the performance of the corresponding effect, i.e., those indicated by the minus signs in Figure 5. Therefore, when the score of the factor *x* is less than that of the factor *y*, a revision of $r_{y}$ is necessary. The revision method we used was based on the concept in [47], where an exponential decay in the form of exp(−RI/C) was used. Here RI is the value of a risk index, and *C* is a constant. Our case is analogous to exponential decay—the coupling coefficient implies a certain risk that may cause the original score to decay. The final equation of score revision is as follows:

(10) $$r_{cy}=r_{y}\cdot \exp\left(\frac{-c_{y\;-\;x}\sqrt{\left(r_{y}-r_{x}+1\right)\cdot r_{y}/r_{x}}}{7}\right)$$

where the difference and quotient between $r_{y}$ and $r_{x}$ together with the constant 7 are determined through the regression, and they are used to scale the coupling coefficient so that we can obtain a reasonable revised score $r_{cy}$. At the current stage, we should clarify that $r_{cy}$ may not be the final revision score of $r_{y}$. This is because there may be several coupling relationships among the same affected factor *y* and different causal factors. For example, “power supply and distribution” is influenced by two causal factors, “fire safety operation standard” and “self-rectification”. Here we assumed that the coupling relationship is independent and they do not affect each other. Therefore, we calculated $r_{cy}$ separately for each coupling relationship and used the lowest revised $r_{cy}$ as the final score. After obtaining all the revised scores ($r_{c}$) for all factors, we obtained the final score for fire safety management with the given normalized weight vectors $w_{norm}^{f}$ of the factors as follows:

$$score=r_{c}\cdot w_{norm}^{f}.$$

**Figure 6:**
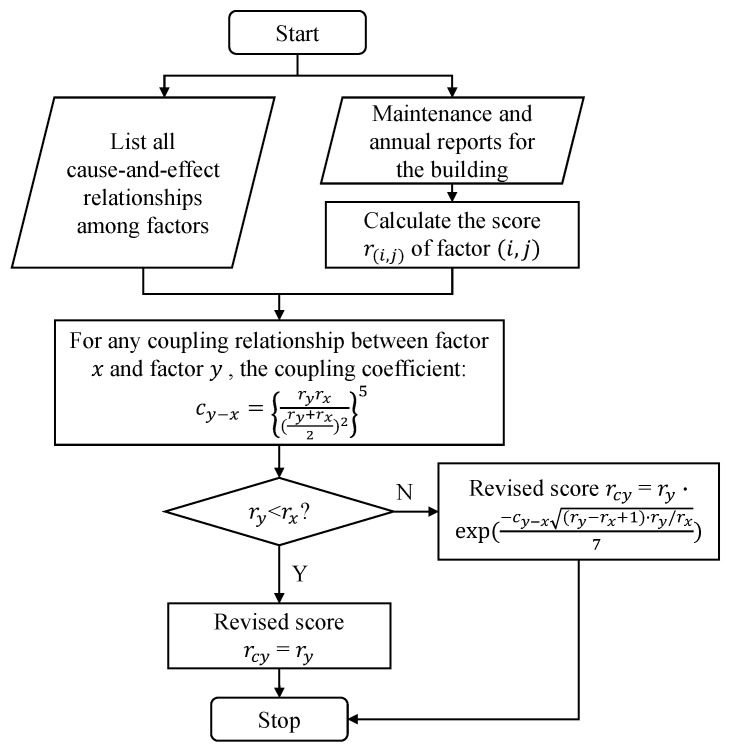
The flow chart of our coupling revision algorithm.

The flow chart of the coupling revision algorithm is shown in Figure 6, and the pseudo-code is given in Algorithm 3.
**Algorithm 3:** Coupling revision.
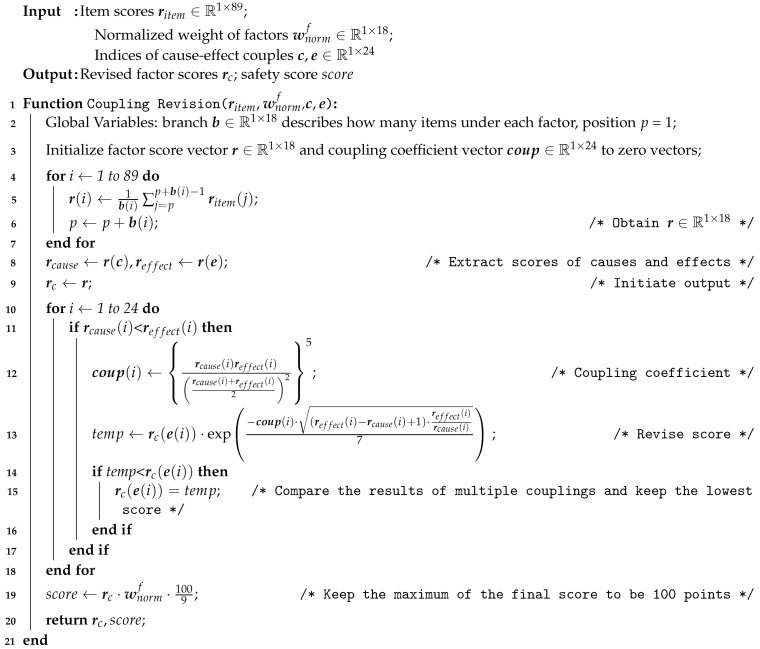


## 4. Experiment and Results

**Figure 7:**
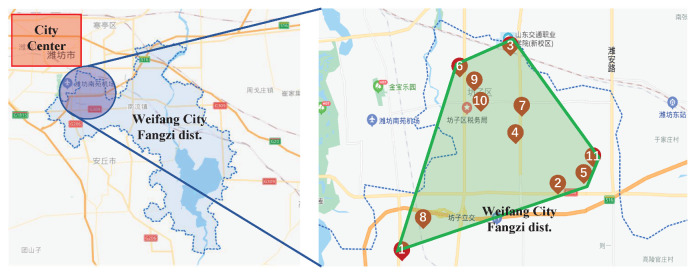
The geographical locations of the 11 buildings.

To validate the proposed evaluation system, we selected 11 large-scale commercial and high-rise buildings in the Fangzi district in Shandong province and collected their score data for all risk factors for analysis. The buildings were labeled 1 to 11, respectively. The name list of those buildings is shown in Table A1. The geographical locations of those buildings are shown in Figure 7. The figure shows that these 11 buildings are densely located in the north-west part of the Fangzi district. Moreover, these 11 buildings are deeply related to the development of the economy, education and science and technology for the whole district. Therefore, the local firefighting body pays lots of attention to the fire risk management of these 11 buildings.

In our experiment, we invited 4 experts to help us evaluate the importance of the categories and the factors in the fire-risk indexing system. Those experts were experienced in the firefighting field. Among them, expert 1 focused on the fire protection water supply system and rescue drills; expert 2 was an experienced practitioner in the maintenance of firefighting facilities; expert 3 participated in the official assessments of fire risk for different kinds of buildings; expert 4 was the minister of the firefighting department in Shandong Province of China, who participated in designing a large number of the national standards related to firefighting.

The experts’ questionnaire results $T_{1}$–$T_{4}$ are summarized as *S* by Equation (1). As for the categories, the overall results are shown in Table 4 according to the questionnaire in Table 2. As for all the factors, the overall results are shown in Table A2. The Roman numerals in those two tables represent the importance scale values (from 1 to 9).

Table 4 and Table A2 in the Appendix A reflect the advantages of using the FAHP. Firstly, we can observe that there is more than one nonzero value in each row, which shows that some answers of the experts are different. In other words, the opinions of the experts on the importance of each category/factor were usually not consistent with one another. The reason is that those invited experts came from different areas, such as fire control, electric power safety and water supply control; some of them were managers, and others inspectors. They tended to concentrate on those categories/factors that were closely related to their daily work. Secondly, for some categories/factors, some experts agreed on the scores; for example, three experts thought that the importance of $F_{1,3}$ was 8. Although the experts were from different areas, their opinions may have converged to a certain extent. Given the above considerations, traditional methods such as the AHP are not fair and objective enough, because the weight distribution is totally decided by only one expert. When the evaluation system is complex enough (such as the one in this work), it is not suitable to consider the opinion of only one expert, since the result is probably biased. For the fire risk evaluation system in this work, the weight distribution is of vital importance, and once distributed well, it will help both the supervision department of the government and the safety chiefs of the buildings to maintain good fire security systems, which will improve public safety. Hence, the weight distribution based on the FAHP is meaningful and has practical value when designing a fire risk evaluation system, since it synthesizes opinions from multiple experts.

Next, we illustrate how to generate the weights of the categories from Table 4. The weights of the factors can be generated similarly. According to Algorithm 1, we first obtained the tuples $\left(l^{\left(i\right)},m^{\left(i\right)},u^{\left(i\right)}\right)$ for each category to summarize the opinions of all the experts as follows:

$$A_{1}:\left(6,9,9\right);A_{2}:\left(7,7,8\right);A_{3}:\left(5,5,9\right);A_{4}:\left(3,6,8\right).$$

Then, the fuzzy matrix *F* and the fuzzy synthetic extent of each category $P_{i}$ were calculated according to Equation (3). The values of the matrix *F* and $P_{i}$ are shown in the first four and last columns of Table 5.

After obtaining the results of fuzzy synthetic extents $P_{i}$, we can compute the comparison matrix $U={\left[\mu_{ab}\right]}_{n\times n}$ of the categories as follows according to the Equation (4):

$$U=\left[\begin{array}{cccc} \infty & 1 & 1 & 1\\ 0.8218 & \infty & 1 & 1\\ 0.5819 & 0.8853 & \infty & 1\\ 0.7176 & 0.9309 & 1 & \infty \end{array}\right].$$

Then, according to the Equations (5) and (6), the normalized weight vector $w_{norm}^{a}$ for the categories can be obtained as:

$$w_{norm}^{a}=\left[\frac{1}{3.1213},\frac{0.8218}{3.1213},\frac{0.5819}{3.1213},\frac{0.7176}{3.1213}\right]=\left[0.3204,0.2633,0.1864,0.2299\right].$$

All the original scores of the factors are collected as r in Table 6. In the same table, the weight of each category ($w_{norm}^{a}$), the weights of the factors in the *i*th category ($w_{norm}^{f,i}$), the original score (r), the revised score of each factor ($r_{c}$) and the final evaluated score (score) of the 11 buildings are shown.

According to $w_{norm}^{a}$ in Table 6, we can see that the category “fire control management” was given the highest weight, followed by “facility maintenance”, “potential risk inspection” and “firefighting and rescue” in descending order. This result is consistent with the importance order given by the categories in Table 6. In a similar way, according to $w_{norm}^{f,i}$, we can also see the order of importance of the factors under each category, which are also consistent with the orders of importance given by the experts. Therefore, our approach is able to assign meaningful weights that are consistent with the opinions of most experts.

The influence of the coupling revision can be validated through the comparison between r and $r_{c}$. Take building 1 as an example. $F_{2,2}$, “automatic fire alarm system”, is coupled with two factors, $F_{1,3}$, “fire safety operation standard”, and $F_{2,10}$, “self-rectification”, according to Figure 5. Since the score of the causal factor $F_{1,3}$ was 9.00, which is larger than that of the affected factor $F_{2,2}$, 7.96, this coupling relationship was ignored. On the other hand, the score of $F_{2,10}$ was only 5.00; hence it undermined the score of $F_{2,2}$. We plugged the scores 5.00 for $F_{2,10}$ and 7.96 for $F_{2,2}$ into Equation (9). Thereby we obtained the coupling coefficient of this cause and effect pair. Then we used this coefficient to revise the original affected score 7.96 to 6.05 by applying Equation (10). The decreasing of the original score means that due to the low evaluation of the causal factor “self-rectification”, the score of the affected factor “automatic fire alarm system” was also undermined. By conducting those revisions, the final score of each factor became more reliable. Now the coupling relationship introduces the influence of external factors into the system, so the revised score can reflect not only the current status of the system, but also the future dynamic trends. For the same factor $F_{2,2}$, we found that another building, number 3, did not revise the score of this factor, and the score remained 7.96 in $r_{c}$. This is because the corresponding two causal factors, $F_{1,3}$ and $F_{2,10}$, had higher scores (both were 9.00) than the affected factor $F_{2,2}$; therefore there was no negative influence on $F_{2,2}$ in those two coupling relationships. Overall, our considerations of all coupling relationships enhanced the building fire risk assessment. For those buildings that did well in all categories, the score revision did not affect them. However, if the building management had any obvious shortcoming, then the shortcoming could have negative influences on other factors. In this way, we improved the strictness of the assessment to a reasonable extent.

## 5. Discussion

In this section, we verify the advantages of the FAHP and coupling revision by comparing our approach with previous ones. The comparison of different approaches had to be based on the same fire-risk indexing system, as shown in Table 1. That is to say, it is unfair to compare approaches based on different inputs. For many existing risk assessment approaches with similar risk indexing systems, the AHP method appears to be widely used [11,35,48]. Therefore, we compared the weight results and safety score results obtained by the AHP and the FAHP using our fire-risk indexing system. In this comparison, all methods applied coupling revision to obtain final safety scores. To further reflect the effect of the coupling revision, we compared the safety scores obtained by using the FAHP with and without coupling revision. To verify the assessment quality, we interviewed relevant parties of those 11 buildings according to the corresponding fire risk assessment results. In addition, in this section, we use an example to illustrate how the assessment scores reflect the reality and will lead the safety chief and the inspectors to improve the fire risk management of each building. We also discuss the possibility of generalizing the proposed approach for other kinds of buildings. At the end, we discuss the future work regarding the proposed approach.

### 5.1. FAHP Performance

**Figure 8:**
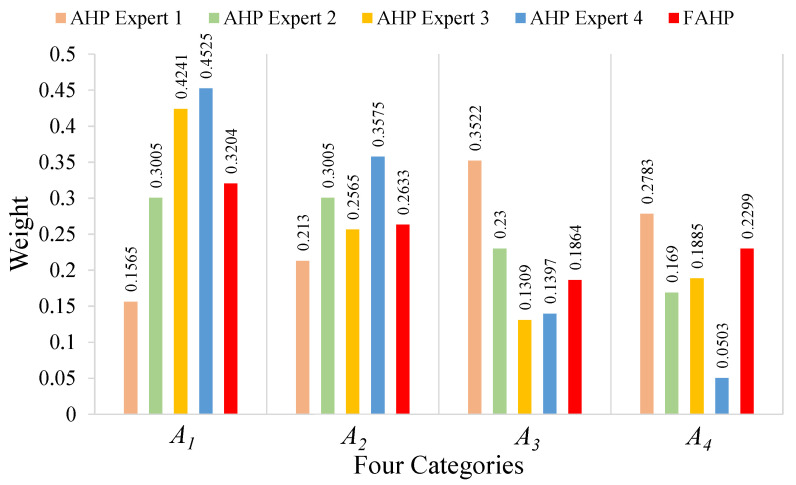
The weights of four categories calculated by FAHP and AHP methods.

To validate the performance of the FAHP, we compared the weights of four categories obtained by the FAHP with those obtained from each expert using the AHP. That is, with the FAHP, we summarized the opinions from all four experts and constructed triangular fuzzy numbers to finally come up with the comparison matrix. With the AHP, a comparison matrix was generated from the opinion of each expert. Therefore, four comparison matrices were generated by the AHP. The five sets of weights are summarized in Figure 8.

**Figure 9:**
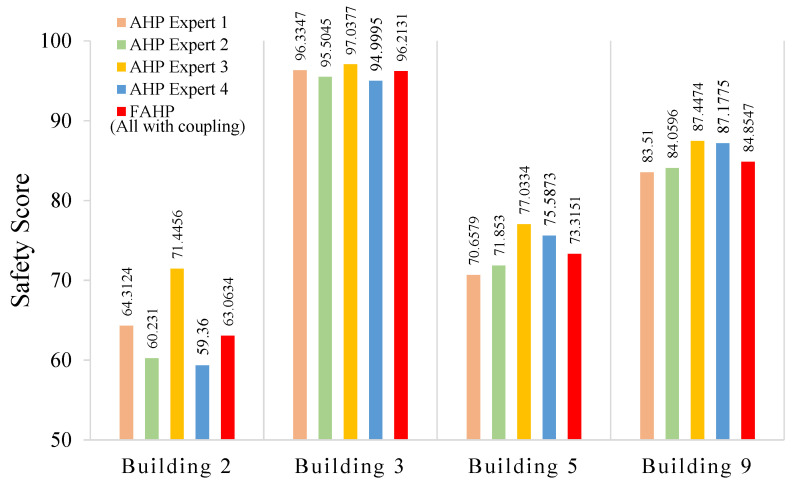
The safety score results obtained by the AHP with coupling revision and the FAHP with coupling revision.

In Figure 8, the numbers over the red bars represent weight results obtained by the FAHP; the others are those obtained by using the AHP. We can see that the weight assignments of the four categories $A_{1}$ to $A_{4}$ varied among different experts; the FAHP method is able to incorporate those differences and come up with a set of more balanced weights. For example, if we only considered the opinion from expert 1, who focused on the water supply system and rescue drills, then $A_{3}$ would have been assigned the highest weight such that his weight distribution would be rather different from those of other three experts. Besides the comparison of weight results, we also conducted the comparison of safety score results obtained by the FAHP and the AHP with coupling revision. We selected four buildings whose safety scores were around 60, 70, 80 and 90, respectively, and summarized the results in Figure 9. The effect of the FAHP on the final safety score was similar to its performance in weight calculations. That is, although the safety scores were quite different among the four experts, the FAHP could combine their results in a scientific way.

### 5.2. Coupling Revision Performance

**Figure 10:**
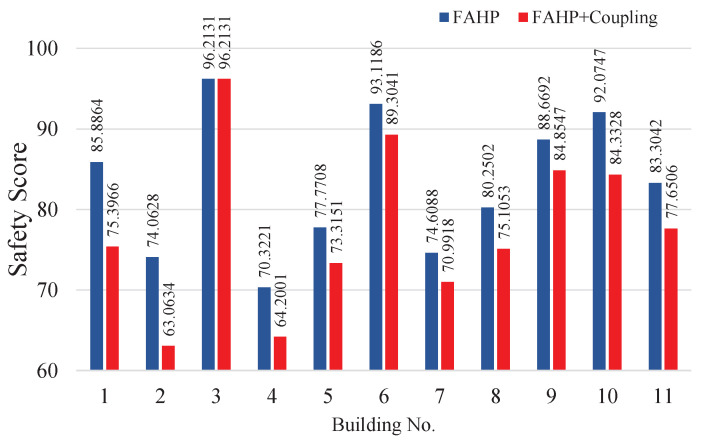
The safety score results obtained by the FAHP with and without coupling revision.

To illustrate the effect of coupling revision, we calculated the safety scores of all 11 buildings using two different methods: the FAHP method without coupling revision and our proposed method with the FAHP and coupling revision. The safety score results of those 11 buildings are summarized in Figure 10. The values over the blue bars are the safety scores obtained by the FAHP without coupling revision; those over the red bars are obtained by the FAHP with coupling revision. The results show that the coupling revision in general dragged down the safety scores, because we consider the negative effect of one factor on another. For example, building 3 has the highest score, 96.2131, which implies that this building does very well in fire safety management, and has almost no deduction from coupling revision. Comparatively, building 2 has a low safety score before coupling revision, and we can observe that the coupling revision pulls down its score from 74.0628 to 63.0634, which reflects that bad performances regarding some factors have negative impacts on other factors. Hence, the addition of coupling revision increases the ability to distinguish among well-managed and ill-managed fire safety systems.

### 5.3. Safety Score Validation and Verification

After evaluating the advantages of FAHP and coupling revision, we use the following example to illustrate how the assessment scores in Table 6 reflect the reality and can lead the safety chiefs and the inspectors to improve the fire risk management of buildings. For building 4, the final score was 64.2001; hence, this building did not perform well in this assessment. There existed several problems in their fire risk management. The following figure lists the weights $w_{norm}^{f}$ of all factors obtained by Equation (8) and the corresponding scores $r_{c}$.

**Figure 11:**
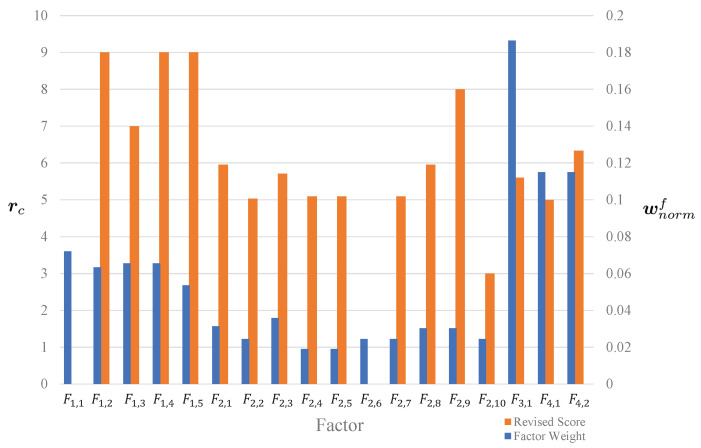
The weight of each factor and the corresponding revised scores for building 4.

From the assessment results of building 4 shown in Figure 11, we can see that $F_{3,1}$ has the highest weight. However, building 4 only got 5.60 points for this factor. In addition, building 4 also did not get high scores in those factors with high weights, such as $F_{4,1}$ and $F_{4,2}$, and it even got 0 points for $F_{1,1}$, which had the fourth highest weight. Therefore, the safety chief of building 4 needed to solve the problems of factors $F_{3,1},F_{4,1}$, $F_{4,2}$ and especially $F_{1,1}$, according to the requirements in the proposed fire-risk indexing system.

Inspectors can record the factors that are important for which a given building gets low scores. Then, when they perform field inspections, the inspectors can easily figure out the corresponding weak points to help the safety chief improve the fire management.

To validate the results obtained by the proposed approach, we present more details about the following factors: $F_{3,1},F_{4,1}$, $F_{4,2}$ and $F_{2,2}$. The original scoring details of the four factors can be found in the annual fire management report and the monthly maintenance report. Original scoring details for other factors are in the similar format. The scoring details for factors $F_{3,1},F_{4,1}$ and $F_{4,2}$ are shown in Table 7. According to the annual fire management report, the scoring of each factor is divided into three discrete ranks. The first rank (I) corresponds to a full score (nine points), and it means that the building fulfills all the requirements regarding this factor. The second rank (II) corresponds to five points, and it represents that the building does well in some of the requirements but still has some defects. The third rank (III) only counts for one point, which means that this factor is unaccounted for in this building. For those factors with zero points, this means that the building does not have any related equipment or documents that can show whether the factor accounted for. According to Table 7, we can see that for factors $F_{3,1},F_{4,1}$ and $F_{4,2}$, most of the items meet the second rank, which matches well with the scores assigned by the proposed approach. The monthly maintenance report provides the original scoring details for $F_{2,2}$, which corresponds to “automatic fire alarm system”. This factor only earned 5.03 points, which is a rather low score. Regarding the maintenance report, the scoring details are listed in Table 8. We can see that the proportion of faulty and abnormal facilities is rather high in the monthly maintenance testing, which shows that the practical performance for this factor is not good.

Other than those low-scoring factors, some factors may have had high scores originally but are revised to lower scores due to the coupling revisions by other factors having low scores. For example, by observing Table 6, factors $F_{2,1}$ and $F_{2,8}$ of building 4 were revised from higher to lower scores. Hence, given the coupling relationship constructed in Figure 5, we can conclude that the proposed revision algorithm can help safety chiefs trace problems back to the original factors which have negative influences on the affected factors. In this way, the safety chiefs can find the most urgent problems for effective improvement.

To verify the assessment quality of the proposed approach, we interviewed those 11 buildings’ chiefs with the corresponding fire risk assessment results. Taking building 4 as an example, according to the assessment results shown in Figure 11, we can see that two factors, $F_{1,1}$ and $F_{2,6}$, obtained zero points. During the investigation, we found that this building had not yet obtained an official certification of fire safety, and it also did not have an emergency service elevator that reached the fire-proof standard. For factors $F_{3,1}$, $F_{4,1}$ and $F_{4,2}$ with high weights and low scores, we also checked the real performance of this building. For factor $F_{3,1}$, the firefighting ability, the safety chief in building 4 organized a volunteer firefighting group from employees, but there was only one record each year of firefighting training and exercise, and most of the volunteers were not good at using the extinguishers. Therefore, the score of firefighting ability was reduced. For $F_{4,1}$, the safety of electricity and gas, the protection for exposed circuits was rather old, and the kitchen flue cleaning was not frequent. These small defects impact fire safety. For $F_{4,2}$, the evacuation facilities, we found that the fire compartment was well designed during the construction, but there were stacks of items along the evacuation passageway, which could obstruct people from evacuation—a severe hidden danger in an emergency. Besides those three factors, the rectification score $F_{2,10}$ was only three points due to the delayed maintenance in this building, which undermined many other factors according to coupling relationships. Overall, building 4 has some essential actual deficiencies, and our score matches its management level.

### 5.4. Instructions for Safety Chiefs and Inspectors

After obtaining the results of the fire risk assessment, our approach can provide feedback that assists the safety chiefs and inspectors with improving fire risk management using the following guidelines:

1. First, our approach presents the weight and the revised score for each factor of the building;
2. Second, according to the weights and revised scores, the factors with relatively low scores and high weights should have high priority for improvements;
3. The factors having low scores and coupled with other factors also should have high priority for improvements.

For building 4, the assessment results are shown in Figure 11 and Table 6. Following the above guidelines, both safety chiefs and inspectors should know the weight and the revised score for each factor of building 4; moreover, they can now know that the factors $F_{3,1}$, $F_{4,1}$ and $F_{4,2}$ had the highest three weights; and factors $F_{1,1}$, $F_{2,6}$ and $F_{2,10}$ had the lowest three revised scores. For both safety chiefs and inspectors, those factors are high priority. If those six factors are rectified, the final assessment score will be 91.0797 points, is 41.87% better.

To be specific, for those factors $F_{3,1},$$F_{4,1}$ and $F_{4,2}$ shown in Table 7, the safety chiefs should rectify the items getting scores less than nine points, and the inspectors should pay attention to those factors in the next assessment. For example, consider factor $F_{3,1}$. The safety chiefs should:

- Design a better firefighting plan which is able to show more details for firefighting drills, such as the escape routes and the fire hydrant boxes;
- Organize a firefighting drill every month or every season; all of the related staff should participate in the drill to practice how to rescue people and how to use the fire extinguishers;
- Check whether the working status of the fire control room satisfies the national standards and quickly rectify the facilities.

As for factor $F_{4,1}$, the safety chiefs should:

- Quickly check the electrical circuits for parts composed of combustible materials—they should be protected with non-flammable materials;
- Clean the kitchen flue at least once every six months and make complete and clear records;
- Carry out inspections on electrical circuits at least once a year, and the inspection report should be submitted to the fire control institution of the local public security organization within three working days from the date of receiving the inspection report.

As for factor $F_{4,2}$, the safety chiefs should:

- Keep the evacuation passageway and the emergency exit clear of obstacles;
- Quickly check the working status of the evacuation facilities and perform regular maintenance.

The inspectors should also pay attention to the above factors to more efficiently check the rectification progress.

Moreover, because the cause and effect coupling relationship is considered in the fire risk assessment, the safety chiefs and inspectors should also focus on those factors with low scores that pull down the scores of the coupled factors. For example, in building 4, the score of $F_{2,1}$, “power supply and distribution”, was revised by $F_{2,10}$, “self-rectification”, from 9 points to 5.95 points. $F_{2,10}$ only got three points because of the terrible rectification performance—most of the faulty facilities were not repaired within a certain time. The large number of faulty facilities probably influenced the power supply in this building. Therefore, $F_{2,10}$ needed high priority for improvement by the safety chiefs, and the inspectors should supervise the rectification progress.

### 5.5. Generalization of the Proposed Approach

For the proposed approach, although it aims to assess the fire safety risks in large-scale commercial and high-rise buildings, it can also be generalized to assess the fire safety risks in other kinds of buildings. When assessing other kinds of buildings, the user of the proposed approach only needs to change the corresponding FRI system. By using a proper FRI system, the safety chiefs and inspectors can generalize a fair and interpretable fire risk assessment for any kind of building. As a comparison, those approaches applying the logistic regression and a deep neural network based on a large amount of historical fire occurrence data for each building type [42] are relatively much harder to generalize due to the difficulty of data collection. Moreover, the proposed approach using the FAHP can combine the knowledge and experience of any number of experts in the field of fire safety or risk assessment without changing any procedure in the approach. As a comparison, those fire risk assessment approaches based on the FRI system [11,35,48], which use the AHP method, can only incorporate the opinion of one expert, making them hard to generalize for complex assessments. In addition, to the best of the authors’ knowledge, the proposed approach is the first to consider the coupling relationship between each pair of factors in the FRI system; those factors are considered separately in other systems. The generalization of the coupling relationship needs to be done once the FRI system is changed to adapt the fire risk assessment approach for other kinds of buildings. For a changed FRI, the coupling relationship shown in Figure 5 should also be updated.

### 5.6. Summary

In summary, there are many studies focusing on building FRI systems to contain more factors so as to conduct better fire risk assessments [11,35,48]. Some new studies without FRI systems apply regression and deep neural networks [42], but it is difficult to collect a large amount of historical data. The place of our work among all this research is that it offers a template for assigning weights to factors in fire risk assessments by combining knowledge and experience from multiple experts based on the risk indexing system. At the same time, the cause and effect coupling relationship between factors is also considered to help both safety chiefs and inspectors find potential weaknesses and improve the efficiency of rectification. Based on this approach, we offered guidelines to guide both safety chiefs and inspectors on how to enhance fire safety management. The proposed approach can intuitively help safety chiefs to improve those factors with low scores and high weights. Hence, they can concentrate on those important factors to enhance the management and save costs on daily maintenance. For inspectors, the proposed approach can also intuitively lead them to improve their efficiency, since they can focus on those factors with bad performances during the assessment. Therefore, according to the above experimental results and the discussion, we can conclude that:

1. The proposed fire risk evaluation system can assign a proper weight to each factor, since the FAHP method is able to combine the opinions on the importance of the categories/factors from multiple experts with different backgrounds in the firefighting field.
2. The proposed approach is designed to be comprehensive and has strong interpretability. The reasons are listed as follows:
  - The proposed approach has a fair and comprehensive indexing system, in which all factors in consideration are fully relative to the fire risk assessment from various perspectives, which captures the features of large-scale commercial and high-rise buildings.
  - In the proposed approach, the scores of some factors are revised if the corresponding coupled factors have bad performances, thereby making the assessment scientific and meaningful.
  - The proposed approach can point out the factors which are important for the assessment when the building gets low scores. Moreover, this approach can find those factors which cause low scores by using the coupling relationships. Therefore, it can intuitively lead both inspectors and the safety chiefs of the large-scale commercial and high-rise buildings to improve their working efficiency, which will be good for public safety.

In the future, we want to construct an intelligent fire risk assessment system that can quickly assess fire safety management and point out real-time weakness of a fire safety system. To this end, we will devote our efforts to combining the firefighting IoT (Internet of Things) data as the inputs to our evaluation system. The reasons for combining IoT data are that: On the one hand, the inputs to the current evaluation system are the monthly maintenance reports and the annual fire management reports from third parties; thus the assessment frequency is rather low. Moreover, the textual documents may have erroneous and even fake data, which might cause trouble during the data processing. On the other hand, firefighting nowadays relies more than ever on the stable and timely data, making manual data collection impossible. The firefighting IoT technology [49,50] can overcome the above-mentioned problems, since the facility data can be collected by sensors all the time. Combining such data into our evaluation system will further improve the quality and efficiency of the current assessment approach.

## 6. Conclusions

In this article, a novel fire risk assessment approach was proposed for large-scale commercial and high-rise buildings. We first designed a fire-risk indexing system based on the Chinese national documents about fire control and management. Then based on this system, we used the FAHP method to integrate all the experts’ knowledge on the importance of each category and factor and distributed weights for the fire risk assessment. In addition, our approach analyzes the coupling relationships among factors in the system and makes necessary revisions for scores of the factors, if necessary. Finally, to validate our assessment approach, we chose 11 large-scale commercial and high-rise buildings in Weifang, Shandong Province, for a case study. The results show that the proposed approach can fairly conduct fire risk assessments. We also interviewed those 11 buildings’ chiefs in regard to the corresponding fire risk assessment results to verify the assessment quality, and we discussed the possibility of generalizing the proposed approach for other kinds of buildings. More importantly, the proposed approach provides both safety chiefs and inspectors with an easy and meaningful way to improve fire risk management. In the future, we will devote our efforts to further integrating the data from IoT systems into our approach to improve fire risk management.

## Figures and Tables

**Table 1:** Fire-risk indexing system.

	Categories	Factors
Fire Risk Factors	Fire Control Management $\left(A_{1}\right)$	Building legitimacy $\left(F_{1,1}\right)$
		Fire safety regulation $\left(F_{1,2}\right)$
		Fire safety operation standard $\left(F_{1,3}\right)$
		Fire prevention patrol $\left(F_{1,4}\right)$
		Publicity and education $\left(F_{1,5}\right)$
	Facility Maintenance $\left(A_{2}\right)$	Power supply and distribution $\left(F_{2,1}\right)$
		Automatic fire alarm system $\left(F_{2,2}\right)$
		Automatic water spraying system $\left(F_{2,3}\right)$
		Smoke exhaust system $\left(F_{2,4}\right)$
		Fire separation facility $\left(F_{2,5}\right)$
		Fire elevator $\left(F_{2,6}\right)$
		Emergency lighting $\left(F_{2,7}\right)$
		Emergency broadcast $\left(F_{2,8}\right)$
		Water supply facility $\left(F_{2,9}\right)$
		Self-rectification $\left(F_{2,10}\right)$
	Firefighting and Rescue $\left(A_{3}\right)$	Firefighting Ability $\left(F_{3,1}\right)$
	Potential Risk Inspection $\left(A_{4}\right)$	Electricity and gas safety $\left(F_{4,1}\right)$
		Evacuation facilities $\left(F_{4,2}\right)$

**Table 2:** Questionnaire template for determining fire risk factor weight distribution. (*M* is the number of categories; $N_{1},N_{2},\cdots ,N_{M}$ represent the number of factors belong to each category.).

Factor/Category	Influence of the Factor/Category								
	1	2	3	4	5	6	7	8	9
Category 1									
Category 2									
⋮									
Category *M*									
	Category 1								
Factor 1									
Factor 2									
⋮									
Factor $N_{1}$									
	Category 2								
Factor 1									
Factor 2									
⋮									
Factor $N_{2}$									
⋮									
	Category *M*								
Factor 1									
Factor 2									
⋯									
Factor $N_{M}$									

**Table 3:** Triangular fuzzy number table.

TFN	Fuzzy Notation	TFN	Fuzzy Notation
(7,9,9)	$\tilde{9}$	$\left(\frac{1}{9},\frac{1}{9},\frac{1}{7}\right)$	$\frac{1}{\tilde{9}}$
(6,8,9)	$\tilde{8}$	$\left(\frac{1}{9},\frac{1}{8},\frac{1}{6}\right)$	$\frac{1}{\tilde{8}}$
(5,7,9)	$\tilde{7}$	$\left(\frac{1}{9},\frac{1}{7},\frac{1}{5}\right)$	$\frac{1}{\tilde{7}}$
(4,6,8)	$\tilde{6}$	$\left(\frac{1}{8},\frac{1}{6},\frac{1}{4}\right)$	$\frac{1}{\tilde{6}}$
(3,5,7)	$\tilde{5}$	$\left(\frac{1}{7},\frac{1}{5},\frac{1}{3}\right)$	$\frac{1}{\tilde{5}}$
(2,4,6)	$\tilde{4}$	$\left(\frac{1}{6},\frac{1}{4},\frac{1}{2}\right)$	$\frac{1}{\tilde{4}}$
(1,3,5)	$\tilde{3}$	$\left(\frac{1}{5},\frac{1}{3},1\right)$	$\frac{1}{\tilde{3}}$
(1,2,4)	$\tilde{2}$	$\left(\frac{1}{4},\frac{1}{2},1\right)$	$\frac{1}{\tilde{2}}$
(1,1,3)	$\tilde{1}$	$\left(\frac{1}{3},1,1\right)$	$\frac{1}{\tilde{1}}$
(1,1,1)	1		

**Table 4:** The overall results for categories from all experts.

	Importance	1	2	3	4	5	6	7	8	9
Category										
$A_{1}$		0	0	0	0	0	I	0	I	II
$A_{2}$		0	0	0	0	0	0	II	II	0
$A_{3}$		0	0	0	0	II	0	I	0	I
$A_{4}$		0	0	I	0	0	II	0	I	0

**Table 5:** The fuzzy matrix *F* and the the fuzzy synthetic extent of the each category $P_{i}$.

	$A_{1}$	$A_{2}$	$A_{3}$	$A_{4}$	$\sum_{b=1}^{n}f_{\mathrm{ib}}$	$P_{i}$
$A_{1}$	(1, 1, 1)	(1, 1, 3)	(1, 2, 4)	(1, 2, 4)	(4, 6, 12)	(0.1333, 0.3529, 1.0141)
$A_{2}$	(0.33, 1, 1)	(1, 1, 1)	(1, 1, 3)	(1, 1, 3)	(3.33, 4, 8)	(0.0111, 0.2353, 0.6761)
$A_{3}$	(0.25, 0.5, 1)	(0.33, 1, 1)	(1, 1, 1)	(0.33, 1, 1)	(1.92, 3.5, 4)	(0.0639, 0.2059, 0.3380)
$A_{4}$	(0.25, 0.5, 1)	(0.33, 1, 1)	(1, 1, 3)	(1, 1, 1)	(2.58, 3.5, 6)	(0.0861, 0.2059, 0.5070)

**Table 6:** The final weights and the revised scores of the categories and factors for the 11 buildings.

No.		Factor/Category in Our Fire Risk System																	
		$A_{1}$					$A_{2}$										$A_{3}$	$A_{4}$	
		$F_{1,1}$	$F_{1,2}$	$F_{1,3}$	$F_{1,4}$	$F_{1,5}$	$F_{2,1}$	$F_{2,2}$	$F_{2,3}$	$F_{2,4}$	$F_{2,5}$	$F_{2,6}$	$F_{2,7}$	$F_{2,8}$	$F_{2,9}$	$F_{2,10}$	$F_{3,1}$	$F_{4,1}$	$F_{4,2}$
	$w_{norm}^{a}$	0.3204					0.2633										0.1864	0.2299	
	$w_{norm}^{f,i}$	0.23	0.20	0.20	0.20	0.17	0.12	0.09	0.14	0.07	0.07	0.09	0.09	0.12	0.12	0.09	1.00	0.50	0.50
1	r	9.00	9.00	9.00	9.00	7.67	6.33	7.96	7.00	7.58	7.00	1.00	9.00	9.00	7.40	5.00	9.00	5.00	7.67
	$r_{c}$	9.00	9.00	9.00	9.00	7.67	5.04	6.05	5.43	5.80	5.43	1.00	6.80	6.80	7.40	5.00	5.69	5.00	7.67
	score	75.3966																	
2	r	0.00	9.00	7.00	9.00	6.33	6.33	7.78	6.08	8.20	5.50	0.00	6.33	9.00	7.36	5.00	8.64	5.00	7.67
	$r_{c}$	0.00	9.00	7.00	9.00	6.33	5.04	4.86	4.89	4.98	4.59	0.00	5.04	5.25	7.36	5.00	5.35	5.00	7.67
	score	63.0634																	
3	r	9.00	9.00	9.00	9.00	9.00	7.00	7.96	6.50	7.80	6.33	9.00	9.00	9.00	8.16	9.00	9.00	8.43	9.00
	$r_{c}$	9.00	9.00	9.00	9.00	9.00	7.00	7.96	6.50	7.80	6.33	9.00	9.00	9.00	8.16	9.00	9.00	8.43	9.00
	score	96.2131																	
4	r	0.00	9.00	7.00	9.00	9.00	9.00	7.19	7.00	6.33	6.33	0.00	6.33	9.00	8.00	3.00	6.60	5.00	6.33
	$r_{c}$	0.00	9.00	7.00	9.00	9.00	5.95	5.03	5.71	5.09	5.09	0.00	5.09	5.95	8.00	3.00	5.60	5.00	6.33
	score	64.2001																	
5	r	9.00	9.00	9.00	9.00	9.00	3.67	6.60	5.00	3.00	6.33	0.00	5.00	1.00	7.40	5.00	8.20	6.33	6.33
	$r_{c}$	9.00	9.00	9.00	9.00	9.00	3.67	5.19	5.00	3.00	5.04	0.00	5.00	1.00	7.40	5.00	6.37	6.33	6.33
	score	73.3151																	
6	r	9.00	9.00	9.00	9.00	9.00	6.33	9.00	7.50	7.18	3.93	5.00	6.33	9.00	7.79	9.00	9.00	9.00	7.67
	$r_{c}$	9.00	9.00	9.00	9.00	9.00	6.33	9.00	7.50	7.18	3.93	5.00	6.33	9.00	7.79	9.00	7.16	9.00	7.67
	score	89.3041																	
7	r	9.00	9.00	7.00	8.00	9.00	6.33	6.60	7.86	7.40	6.33	0.00	7.67	0.00	8.20	5.00	5.80	6.60	6.33
	$r_{c}$	9.00	9.00	7.00	8.00	9.00	5.04	5.19	4.88	4.78	5.04	0.00	4.83	0.00	8.20	5.00	5.80	6.60	6.33
	score	70.9918																	
8	r	9.00	9.00	9.00	9.00	9.00	5.00	9.00	5.00	0.00	7.67	0.00	6.33	0.00	7.50	5.00	9.00	5.00	7.67
	$r_{c}$	9.00	9.00	9.00	9.00	9.00	5.00	6.80	5.00	0.00	5.85	0.00	5.04	0.00	7.50	5.00	7.16	5.00	7.67
	score	75.1053																	
9	r	9.00	9.00	9.00	9.00	7.67	6.33	7.94	7.67	8.20	7.00	5.00	9.00	5.00	7.00	9.00	9.00	5.00	9.00
	$r_{c}$	9.00	9.00	9.00	9.00	7.67	6.33	7.94	7.67	8.20	7.00	5.00	9.00	5.00	7.00	9.00	7.16	5.00	9.00
	score	84.8547																	
10	r	9.00	9.00	9.00	9.00	9.00	6.33	8.20	8.43	9.00	9.00	9.00	9.00	5.00	8.27	5.00	9.00	7.29	7.67
	$r_{c}$	9.00	9.00	9.00	9.00	9.00	5.04	6.22	6.38	6.80	6.80	6.80	6.80	5.00	8.27	5.00	7.16	7.29	7.67
	score	84.3328																	
11	r	9.00	9.00	9.00	9.00	9.00	5.00	8.05	7.50	5.80	7.67	0.00	7.67	0.00	7.06	5.00	8.60	6.33	7.67
	$r_{c}$	9.00	9.00	9.00	9.00	9.00	5.00	6.11	5.74	4.74	5.85	0.00	5.85	0.00	7.06	5.00	6.99	6.33	7.67
	score	77.6507																	

**Table 7:** Scoring details of factors $F_{3,1},F_{4,1}$ and $F_{4,2}$ in the annual fire management report.

	Assessment Item	I(9’)	II(5’)	III(1’)
$F_{3,1}$	Firefighting plan		✓	
	Firefighting drill		✓	
	Fire extinguisher	✓		
	Volunteer fire-fighting group	✓		
	Fire control room		✓	
$F_{4,1}$	Power management	✓		
	Electrical circuit protection		✓	
	Kitchen flue cleaning		✓	
	Annual electricity inspection			✓
$F_{4,2}$	Evacuation passageway and emergency exit		✓	
	Fire compartment	✓		
	Evacuation facilities		✓	

**Table 8:** Scoring details of factor $F_{2,2}$ in the maintenance report.

		Facilities Tested	Normal	Faulty
Automatic fire alarm system $F_{2,2}$	Heat fire detector	9	8	1
	Photoelectric smoke fire detector	31	28	3
	Fire display panel	34	0	34
	AC power failure and recovery	1	0	1
	Standby power failure and recovery	1	1	0

## Data Availability

The study did not report any data.

## Appendix A

**Table A1:** The name list of the buildings used in the experiments.

No.	Name of the Buildings	Area (m${}^{2}$)	Height (m)	Storey
1	Shandong Hua ’an Automobile Culture Development Co. Ltd.	27,202.25	23.95	4
2	Shandong Huakai Bike Wire Harness Co. Ltd.	16,957.1	15.3	3
3	Shandong Transportation Vocational College	12,302.9	41.1	11
4	Fangzi District Financial Huitong Hotel	18,931	64.8	17
5	Shandong Jingbei Fintech Co. Ltd.	18,254	19.2	4
6	Levo Heavy Industries Co. Ltd.	14,600	38.4	10
7	Longfeng Garden Community	9347.24	36	12
8	Shandong Luneng Taishan Soccer School	2703.36	16.5	3
9	Rural Commercial Bank Co., Ltd. Fangzi Sub-branch	6400	41.2	10
10	Fang Zi Chamber of Commerce Building	26,921.86	49.95	17
11	Shandong Xingang Electronic Technology Co. Ltd.	18,254	19.2	4

**Table A2:** The overall results for factors from all experts.

	Importance	1	2	3	4	5	6	7	8	9
Factor										
$F_{1,1}$		0	0	I	0	0	I	0	0	II
$F_{1,2}$		0	0	0	0	0	0	II	I	I
$F_{1,3}$		0	0	0	0	0	0	0	III	I
$F_{1,4}$		0	0	0	0	0	I	0	III	0
$F_{1,5}$		0	0	0	0	0	II	I	I	0
$F_{2,1}$		0	0	I	0	0	0	II	0	I
$F_{2,2}$		0	0	0	0	I	I	0	I	I
$F_{2,3}$		0	0	0	0	0	0	I	0	III
$F_{2,4}$		0	0	0	I	I	0	I	I	0
$F_{2,5}$		0	0	0	I	I	I	0	I	0
$F_{2,6}$		0	0	0	0	I	I	I	I	0
$F_{2,7}$		0	0	0	I	II	I	0	0	0
$F_{2,8}$		0	0	0	I	0	II	I	0	0
$F_{2,9}$		0	0	0	0	0	I	I	I	I
$F_{2,10}$		0	0	0	0	I	I	0	I	I
$F_{3,1}$		0	0	0	I	I	0	I	0	I
$F_{4,1}$		0	0	0	0	I	0	0	II	I
$F_{4,2}$		0	0	0	0	I	0	0	II	I
